# Silica-Bacterial Cellulose Composite Aerogel Fibers with Excellent Mechanical Properties from Sodium Silicate Precursor

**DOI:** 10.3390/gels8010017

**Published:** 2021-12-26

**Authors:** Qiqi Song, Changqing Miao, Huazheng Sai, Jie Gu, Meijuan Wang, Pengjie Jiang, Yutong Wang, Rui Fu, Yaxiong Wang

**Affiliations:** 1 School of Chemistry and Chemical Engineering, Inner Mongolia University of Science & Technology, Baotou 014010, China; songqiqiaa@163.com (Q.S.); qingmc@163.com (C.M.); gujie199504182021@163.com (J.G.); wmjbest1014@163.com (M.W.); jpj1692787089@163.com (P.J.); wangyut@163.com (Y.W.); wangyaxiong2021@126.com (Y.W.); 2 Inner Mongolia Engineering Research Center of Comprehensive Utilization of Bio-Coal Chemical Industry, Inner Mongolia University of Science & Technology, Baotou 014010, China; 3 Aerogel Functional Nanomaterials Laboratory, Inner Mongolia University of Science & Technology, Baotou 014010, China

**Keywords:** fibrous aerogel, secondary shaping, mechanical properties, thermal properties, environmentally friendly

## Abstract

Forming fibers for fabric insulation is difficult using aerogels, which have excellent thermal insulation performance but poor mechanical properties. A previous study proposed a novel method that could effectively improve the mechanical properties of aerogels and make them into fibers for use in fabric insulation. In this study, composite aerogel fibers (CAFs) with excellent mechanical properties and thermal insulation performance were prepared using a streamlined method. The wet bacterial cellulose (BC) matrix without freeze-drying directly was immersed in an inorganic precursor (silicate) solution, followed by initiating in situ sol-gel reaction under the action of acidic catalyst after secondary shaping. Finally, after surface modification and ambient drying of the wet composite gel, CAFs were obtained. The CAFs prepared by the simplified method still had favorable mechanical properties (tensile strength of 4.5 MPa) and excellent thermal insulation properties under extreme conditions (220 °C and −60 °C). In particular, compared with previous work, the presented CAFs preparation process is simpler and more environmentally friendly. In addition, the experimental costs were reduced. Furthermore, the obtained CAFs had high specific surface area (671.3 m²/g), excellent hydrophobicity, and low density (≤0.154 g/cm^3^). This streamlined method was proposed to prepare aerogel fibers with excellent performance to meet the requirements of wearable applications.

## 1. Introduction

Aerogels are solid substances that possess many excellent properties such as high specific surface area (500–1200 m^2^/g), high porosity (80–99.8%), low density (~0.003 g/cm^3^), low thermal conductivity (0.005 W/m·K), and optical transparency [1,2,3]. Based on these favorable characteristics, aerogel products, which are considered to be among the most promising nanomaterials, are mainly used in adsorption, catalysis, heat insulation, biomedicine, oil spill cleaning, and other fields [4,5].

Currently, from the point of view of material composition, aerogels can be classified into the following categories: inorganic oxide aerogels (e.g., SiO_2_ aerogels [6], TiO_2_ aerogels [7], Al_2_O_3_ aerogels [8]), organic aerogels (e.g., cellulose aerogels [9], polyurethane aerogels [10]), carbon aerogels (e.g., graphene aerogels [11], carbon nanotube aerogels [12]), and other aerogels (e.g., metal aerogels [13]). Aerogels are not only rich in material composition, but also have various microstructures of the gel skeleton. The building blocks of the aerogel gel skeleton mainly include: (i) zero-dimensional (0D) nanoparticles [14], (ii) one-dimensional (1D) nanowires [15], and (iii) two-dimensional (2D) nanosheets [16]. Different element compositions and microstructures cause aerogels to exhibit various excellent properties for different applications.

Thermal insulation is the most noticeable feature of aerogels. Because the porosity of aerogels can be as high as 90% [3], the skeletons of the aerogels are filled with gas. Furthermore, the pore size of aerogel nanoscale (< 50 nm) [17] is lower than the average free path of air molecules (approximately 70 nm), which does not satisfy the condition of free gas flow, in which the gas molecules pass through the pores of the aerogels without being subjected to collisions and do not exchange energy with each other [18,19,20]. In addition, the gel skeleton of aerogels is a three-dimensional nanowork composed of nano elements, which increases the heat transfer path when the heat passes through the gel skeleton, thus effectively limiting the thermal conduction. Therefore, the macroscopic thermal conductivity of aerogel material is in an extremely low range [21]. In particular, the heat transfer path of gel skeletons composed of 0D nanoparticles increased more obviously than that of gel skeletons composed of 1D nanofibers and 2D nanosheets. In addition, the limited connection area between nanoparticles also makes it particularly difficult for the gel skeleton to transfer heat. Therefore, aerogels composed of nanoparticles have better thermal insulation properties than the others [22].

Although the limited connection area between nanoparticles gives aerogels excellent thermal insulation, it leads to poor mechanical properties. For example, most typical silica aerogels [23], which are the earliest and most widely studied type of aerogels among inorganic oxide aerogels, are extremely brittle, making them difficult to use alone. The microstructure of the silica gel skeleton is similar to a “pearl necklace” [24]. The connection point between the silica secondary particles aggregated in the three-dimensional network is very fragile, which causes the silica aerogels to exhibit brittleness, making them difficult to be processed as fibers and applied in the field of fabric insulation. Hence, silica aerogels can be easily broken into finer particles [6].

A significant amount of research has been conducted to improve the mechanical properties of silica aerogels. Generally speaking, there are two ways to enhance the mechanical properties of aerogels [25]. One is to enhance the strength of the gel skeleton by regulating the precursor molecular structure or applying polymer isomorphic coating [26]. For example, epoxy resin [27] and triisocyanate [28] can be used as reinforcing agents to coat the junction between aerogel nanoparticles to enhance the strength of the aerogel gel skeleton. The other is to develop composite aerogel products [29]. Fibrous cellulose [30], fiberglass [22], and aramid [25] were used to reinforce the silica gel skeleton to produce composite aerogel products with high mechanical properties. The above work has improved the mechanical properties of aerogels to a certain extent, while it is still not satisfactory. For example, powder shedding often occurs during the use of aerogel felt [31]. Therefore, to enrich the existence form of aerogels (aerogel fibers) and meet the requirements of different fields, it is necessary to further improve their mechanical properties.

**Figure 1:**
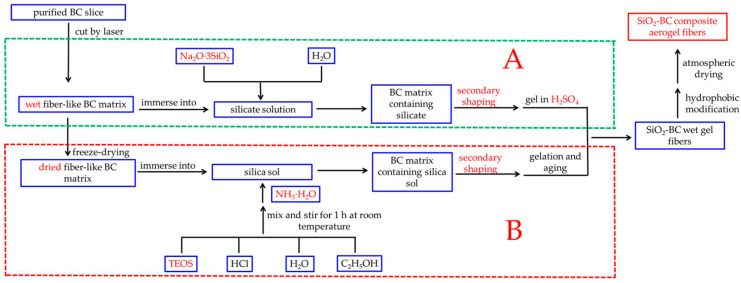
Flowchart illustrating the overall processes used in this work (**A**) and previous work (**B**).

Recently, researchers have performed very fruitful work on the synthesis of aerogel fibers. Aerogel fibers can be prepared by direct spinning and indirect molding. Direct spinning, which includes freeze-spinning and wet-spinning, is directly accomplished by adjusting the gel process. Yu Du et al. [32] elaborately controlled the ratio of the spinning solution and coagulation bath to achieve rapid gelation to obtain a continuous reaction-spinning of transparent silica aerogel fibers. Meng Si et al. [33] controlled and studied the hierarchical structure and hollow structure of silica aerogel fibers by changing the sulfuric acid concentration of the aging bath and the spinning speed. Although the direct spinning process is relatively simple, the mechanical properties of the prepared aerogel fibers are not satisfactory. Indirect molding is more complex and is mainly used for the preparation of composite aerogel fibers with special structures. For example, by first preparing a cellulose acetate/polyacrylic acid (CA/PAA) hollow fiber using coaxial wet-spinning followed by injecting the silk fibroin (SF) solution into the hollow fiber, CA/PAA-wrapped SF aerogel fibers for textile thermal insulation were successfully constructed after freeze-drying [19]. Jian Zhou et al. [34] successfully engineered multiscale porous acetate/polyacrylic acid (CA/PAA) sheath and cellulose nanofibril (CNF) aerogel core as novel thermal insulation composite aerogel fibers with high porosity. Although the mechanical properties of the composite aerogel fibers have been greatly improved, in indirect molding, hollow fibers and an internal gel skeleton are prepared, in sequence. Therefore, the preparation process is complex and time-consuming. Hence, in our earlier study [35], a new method for preparing aerogel fibers was proposed (part B in Figure 1). This method involves: immersing the dried fibrous bacterial cellulose (BC) matrix which is a green, natural, and degradable organic polymer with excellent mechanical properties into the silica sol, followed by immediately passing the BC matrix containing silica sol through a conical mold to make the fibrous BC matrix finer and more uniform. This process, called secondary shaping, significantly increases the content of BC nanofibers per unit volume of the matrix. In addition, compared with cellulose extracted from plants, the purification of BC formed by microbial fermentation is easier to achieve. More importantly, BC has higher crystallinity, so it has better mechanical properties [36,37]. Therefore, the obtained aerogel fibers exhibit excellent mechanical properties. However, this method also has the following disadvantages: (i) the gel process is highly susceptible to temperature, so it is difficult to ensure that the silica precursor can fully diffuse into the matrix before the gel; (ii) the process of freeze-drying the BC matrix takes several hours, which makes it too time-consuming to scale up production; and (iii) the silica source used in the experiment was TEOS, which is environmentally unfriendly and expensive.

In this paper, a streamlined method for preparing silica-cellulose composite aerogel fibers (CAFs) is proposed (part A in Figure 1). A long strip (without freeze-drying), which was soaked in silica precursor solution, was cut from cellulose hydrogel using a laser cutter. Then, the resulting material was passed through a tapered mold to regulate the matrix morphology at both the macroscopic and microscopic levels through secondary shaping. Finally, silica-cellulose composite aerogel fibers were obtained through hydrophobic modification and ambient drying. We further optimized the experimental process from our previous work, using Na_2_O·3SiO_2_ instead of TEOS as the silica source; furthermore, wet BC matrix was used in the experiment, so the freeze-drying step was omitted. In particular, this gel process was simple and easy to control. This improvement not only reduced the cost, but also made the synthesis process more environmentally friendly. More importantly, this improvement simplified the operation and saved time. Because of this improvement, the CAFs still have excellent mechanical properties; in addition, this development promotes the application of aerogels in fabric insulation. 

## 2. Results and Discussion

### 2.1. Diffusion of Silicate Solution in Fiber-like BC Matrix

**Figure 2:**
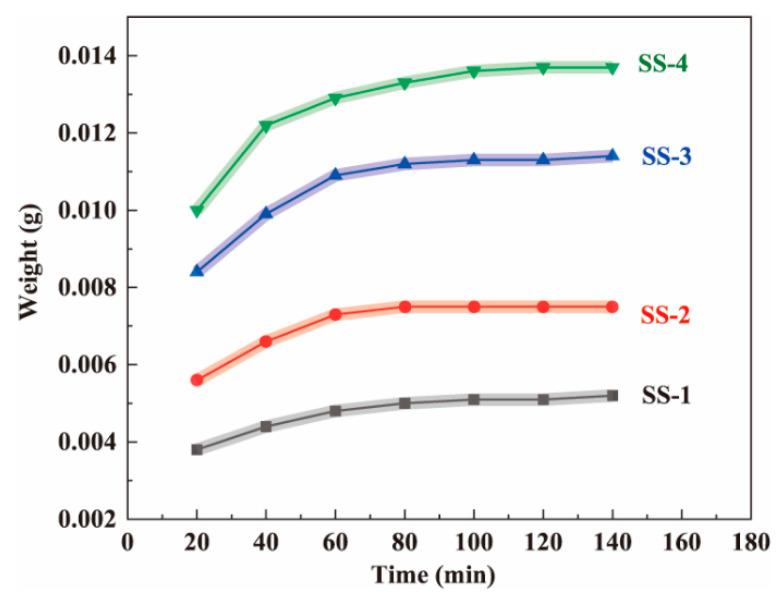
The effect of soaking time in the sodium silicate solutions (SS-1, SS-2, SS-3, and SS-4, respectively) on the weight of the dried BC wet gel samples.

Each wet fiber-like BC matrix (8 cm long) was immersed in the silicate solutions (SS-1, SS-2, SS-3, and SS-4) at room temperature for varying amounts of time [38]. Then, the samples that had been soaked for different times were placed in an oven to dry (at 80 °C for 20 min). In the above process, the silica precursor prediffused into the BC matrix will not fall from the BC nanofiber network. Figure 2 shows that the higher the solution concentration, the higher the sample mass. By weighing the weight of the dried samples, it was found that the weight of the samples initially increased rapidly, then tended to decelerate over time, and barely increased after 80 min. The experimental results showed that silicate could diffuse into the wet fiber-like BC matrix and reach diffusion equilibrium at approximately 80 min. In addition, after reaching the diffusion equilibrium, the higher the concentration, the greater the sample mass, indicating that more silica precursors entered the matrix. Therefore, compared with the previous experiment, freeze-drying the wet fiber-like BC matrix, which was a time-consuming and energy-consuming step, was avoided. Meanwhile, based on this experimental result, the time required for the silica precursor to reach diffusion equilibrium in the wet fiber-like BC matrix was determined. Therefore, the wet fiber-like BC matrix immersion time was determined to be 2 h in subsequent experiments to ensure full diffusion.

### 2.2. Wettability

**Figure 3:**
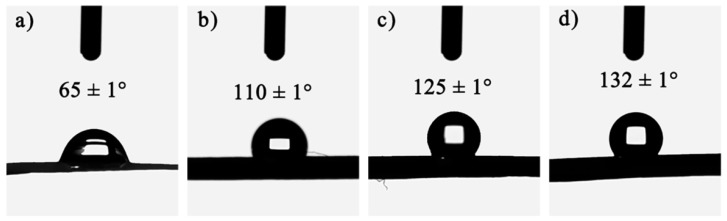
Wettability of CAF-1 (**a**), CAF-2 (**b**), CAF-3 (**c**), and CAF-4 (**d**). (The sample number is shown in Table 1).

To ensure that the gel skeleton can spring back during the ambient drying process and prevent water vapor from damaging the nanopore structure of the CAFs, hydrophobic modification (changing hydrophilic hydroxyl groups into hydrophobic alkyl groups) of the wet gel fibers was carried out. The wettability of the samples was then investigated. As shown in Figure 3, with the increase in the amount of silica precursor in the samples, the contact angle of the samples also increased significantly. Moreover, CAF-2, CAF-3, and CAF-4 exhibited excellent hydrophobicity. There were two reasons for this phenomenon. One was that with the increase in silica precursor concentration, the silica content on the outer surface of the CAFs gradually increased, so that the outer surface roughness of the CAFs also increased. The other was that the outer surface of the CAFs had both silica hydroxyl and carbon hydroxyl. Silica hydroxyl was more active and reacts more easily with TMCS, so the higher the silica content, the more hydrophobic alkyl groups there were on the outer surface after hydrophobic modification. Therefore, the increase in hydrophobic groups and the increase in outer surface roughness resulted in CAFs with better hydrophobicity. However, compared with our previous work, the hydrophobicity of the CAFs decreased slightly. This was because during the gel process, the silica in the BC matrix also diffused into the sulfuric acid solution, resulting in a decrease in the silica content on the CAF surface, resulting in a decrease in hydrophobicity.

### 2.3. Microstructures

**Figure 4:**
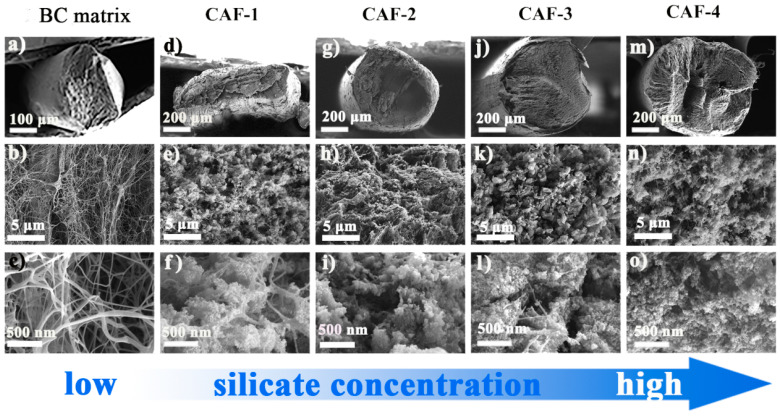
SEM images of BC matrix and CAFs with different magnifications ((**a**–**c**) were SEM images of BC matrix enlarged by 50×, 5000× and 20,000×. (**d**–**f**), (**g**–**i**), (**j**–**l**) and (**m**–**o**) were SEM images of CAF-1, CAF-2, CAF-3 and CAF-4 enlarged by 100×, 5000× and 20,000×, respectively).

As shown in the SEM images (Figure 4), compared with the BC matrix, the silica gel skeleton was found in all CAFs. The diameter of CAF-4 (Figure 4m) was close to the inner diameter of the tapered mold, which indicated that there was little shrinkage during the ambient drying process, which meant that the gel skeleton could spring back effectively during ambient drying. However, compared with CAF-4, CAF-2 and CAF-3 shrunk slightly with the decrease in silica precursor concentration, and CAF-1 shrunk so severely that its cross section was no longer circular. This phenomenon could be explained by the fact that when the concentration of silica precursor was relatively low, there was not enough gel skeleton inside the aerogel fibers or the gel skeleton was not robust enough to resist the capillary force of the samples, resulting in the collapse of the gel skeleton and the shrinkage of the samples during ambient drying. In addition, during the ambient drying process of CAFs, the more serious shrinkage of the silica gel skeleton with a lower silica precursor concentration led to an increase in the density of the silica gel skeleton in the unit volume at the micro level, which resulted in similar microstructures of CAF-1, CAF-2, and CAF-3 (Figure 4e–k). In contrast, CAF-4 had the highest content of silica precursor, the silica gel skeleton was more compact, and fiber filaments were hardly visible in the field of vision. The infrared spectroscopy analysis also showed that the composite aerogel fibers contained a large number of gel skeletons (Figure S4).

**Figure 5:**
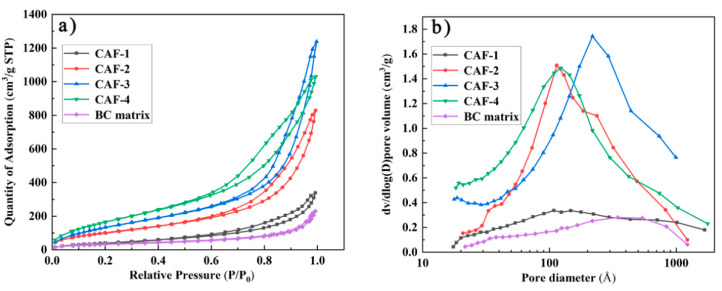
Nitrogen adsorption–desorption (**a**) isotherms and pore size distribution (**b**) of CAFs.

To further investigate the pore characteristics of the obtained CAFs, N_2_ adsorption-desorption isotherms of the samples were measured. The hysteresis loops which existed in the nitrogen adsorption-desorption isotherm were type IV isotherms (Figure 5a) of the prepared CAFs, confirming that the prepared samples contained a mesoporous structure. However, the nitrogen adsorption-desorption isotherm and pore size distribution confirmed that there was almost no mesoporous structure in the BC matrix. This phenomenon indicated that the existence of the silica gel skeleton could provide a mesoporous structure for CAFs. As shown in Figure 5a, the hysteresis loops of CAF-2, CAF-3, and CAF-4 were more obvious than those of CAF-1. Furthermore, the pore size distribution (Figure 5b) showed that the mesoporous structure of CAF-1 was the least obvious. Finally, a BET test showed that the specific surface area of the samples increased from 158.6 to 671.3 m²/g (Table 1) with the increase in silica precursor concentration. This was because CAF-1 had the lowest silica precursor content, and most of the nanoparticles were stacked together or simply adhered to the matrix without forming an effective three-dimensional network structure. Therefore, the mesoporous structure of CAF-1 was the least obvious. These phenomena indicate that the concentration of silica precursor has a remarkable effect on the pore structure of CAFs, and it is essential to control the level of the precursor to obtain the most suitable microstructure.

### 2.4. Mechanical Properties

**Figure 6:**
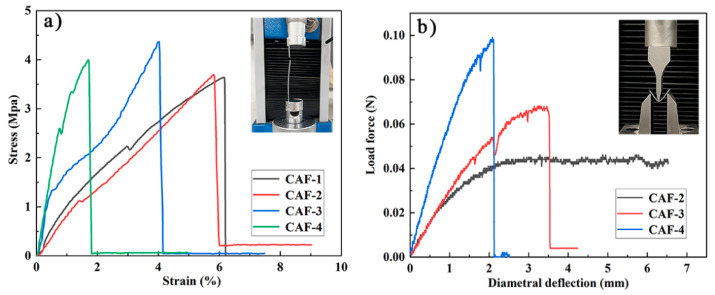
Stress-strain curves of tensile tests (**a**) and three-point bending tests (fixture span is 15 mm) (**b**) of CAFs.

Mechanical properties are crucial to determine whether aerogel materials can be prepared as aerogel fibers and applied in the field of fabric insulation. A tensile test and three-point bending test were carried out to clarify the mechanical properties of the prepared CAFs. The mechanical performance of CAFs prepared with different concentrations of Na_2_O·3SiO_2_ precursor is shown in Figure 6. Figure 6a shows that all samples exhibited excellent tensile strength. The tensile strength of the CAFs was 3.5–4.5 MPa, which was much higher than that of native silica aerogel fibers, such as SiO_2_ aerogel fibers (230 KPa). It is worth mentioning that the tensile strength of the CAF-3 was approximately 4.5 MPa, which is higher than that of some organic aerogel fibers, such as CA/PAA-SF aerogel fibers (approximately 2.6 MPa), CA/PAA-SF/GO aerogel fibers (approximately 3.0 MPa), and QF/ASA aerogel fibers (approximately 3.17 MPa) (Table 2). This further confirmed the excellent mechanical properties of the aerogel fibers prepared by compounding cellulose and silica. In addition, the stress-strain curves of CAFs also showed that the elongation at break of the samples decreased from 6.1% to 1.8% with increasing silica precursor concentration. The same phenomenon also occurred in the three-point bending test. CAF-4, which had the highest silica precursor content, had less deformation than CAF-3, which had relatively small silica precursor content; furthermore, CAF-2, which had lower silica precursor content, had no obvious fractures in a large deformation range (Figure 6b and Video S1). This phenomenon could be explained by the assumption that as the concentration of the silica precursor gradually increased, more and denser gel skeletons formed between the BC nanofibers, restricting the free movement of nanofibers, resulting in the free deformation space of the nanofibers being compressed. In addition, the stress required for CAF-3 and CAF-4 fractures also increased with an increase in the silica precursor content in the three-point bending test. This phenomenon demonstrated that with an increase in silica precursor concentration, the increase in gel skeletons resulted in higher rigidity and enhanced the ability of CAFs to resist external impact; however, it resulted in higher brittleness in the CAFs. Therefore, controlling the concentration of the silica precursor is critical for preparing aerogel fibers with excellent mechanical properties.

### 2.5. Thermal Insulation

**Figure 7:**
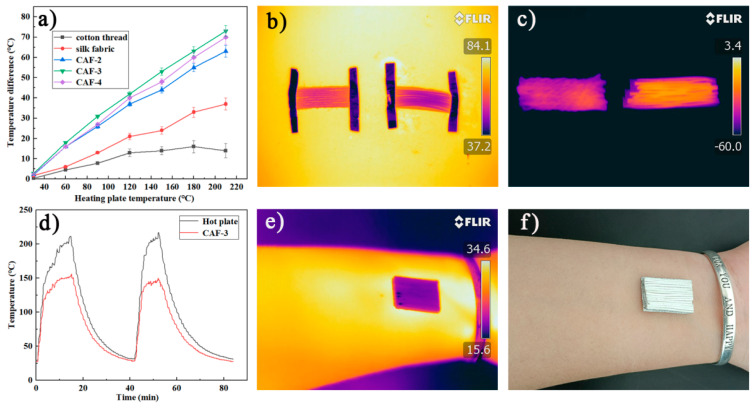
Thermal insulation properties of CAFs, silk fabric, and cotton threads. Temperature difference between the fiber surface and hot plate versus temperature of the hot plate for the single-layer mats made of CAFs, silk fabric, and cotton threads (**a**). Infrared images of one-layer mats of CAF-3 and cotton threads at high and low temperatures (**b**,**c**). Temperature-time curves of CAF-3 and hot plate (**d**). Infrared images and optical images of the CAF-3 fabric covered on a human arm for the room-temperature thermal insulation test (**e**,**f**).

Cotton threads and silk fabric with similar diameters and thicknesses to those of CAFs were tested for insulation under the same conditions. Figure 7a showed that the |∆T| of a one-layer CAF mat was more efficient than a one-layer silk fabric mat and cotton fabric mat in terms of thermal insulation. When T_h_ = 150 °C, the temperature of the CAF mat was 100 °C, while the surface temperature of the cotton thread mat and silk fabric mat reached 136 °C and 126 °C, respectively, indicating that the CAFs had better thermal insulation properties than cotton threads and silk fabric. In order to highlight the excellent thermal insulation performance of CAFs, we compared the temperature changes of a one-layer CAF (CAF-3) mat and a one-layer cotton fabric mat at high (80 °C) and low (−60 °C) temperatures. After the temperature became stable, a series of infrared images were taken. Figure 7b,c show that the CAFs demonstrated excellent thermal insulation performance at high (80 °C) and low (−60 °C) temperatures.

According to Figure 7a, with the increase in silica precursor concentration, the thermal insulation performance of the corresponding CAF-2 and CAF-3 gradually improved, because more and denser gel skeletons were formed with the increase in the solid concentration in the aerogels. However, CAF-4, which had the highest concentration, did not match this trend, because the solid content of CAF-4 was too high; heat was more likely to transfer along the solid phase, resulting in inferior insulation performance [38,42]. To further investigate the stability of the CAF insulation performance, the dynamic temperature changes on the surface of the hot plate (T_h_) and aerogel fibers (CAF-3) during the heating-cooling cycle (Figure 7d) were evaluated. The surface temperature of CAF-3 varied from 25 °C to 150 °C, while the temperature of the hot plate increased from 25 °C to 220 °C (Figure S3 shows that the samples still have excellent stability at this temperature). Moreover, with an increase in the temperature of the hot plate, |ΔT| also increased. When temperature of the hot plate (T_h_) reached more than 200 °C, the temperature of CAF-3 remained at approximately 150 °C and did not change significantly. When CAF-3 was heated again after a heating-cooling process, the |ΔT| of the hot plate and CAF-3 showed no obvious change, indicating that the thermal insulation performance of CAF-3 was stable.

The thermal insulation performance of multilayer CAF-3 was also investigated (Figure S2). The experimental results showed that the higher the number of layers, the better the thermal insulation performance. For the three-layer CAF-3 fabric, |ΔT| is as high as 125 °C on a hot plate at 210 °C, which is approximately 15 °C and 55 °C higher than the corresponding values for the two-layered and one-layered CAF-3 fabric, respectively. Meanwhile, in order to evaluate the thermal insulation properties of CAF-3 at natural temperature, CAF-3 fabric was attached to human skin (Figure 7f). The infrared thermal image (Figure 7e) showed that the surface temperature of the CAF-3 fabric was close to the background temperature, indicating that CAF-3 fabric may be used as a thermal stealth material in the future.

## 3. Conclusions

In conclusion, composite aerogel fibers (CAFs) with excellent mechanical properties and thermal insulation performance were prepared by directly diffusing sodium silicate solution into wet BC matrix, followed by an in situ sol-gel reaction under the action of an acidic catalyst. Owing to the loose microstructure and gelation rate of the BC nanofiber matrix, the silica precursor effectively diffused into the BC matrix within 2 h, then formed a silica gel skeleton in the BC matrix. In contrast to previous work, Na_2_O·3SiO_2_ was used as the silica source instead of TEOS as silica source, thus lowering costs, and the previous drying of the wet BC matrix was found to be unnecessary. The CAFs exhibited low density (≤0.154 g/cm^3^), high porosity (≥80.3%), and high specific surface area (>670 m^2^/g), as well as excellent mechanical properties. The mechanical properties were easily improved by the secondary shaping, which significantly increased the content of BC nanofibers per unit volume of the BC matrix. This increased the breaking stress to 4.5 MPa. Above all, these CAFs have excellent thermal stability and are hydrophobic, which enables them to be used in harsh environments and expands the range of use of aerogels.

## 4. Materials and Methods

### 4.1. Materials

Nata-de-coco slices were purchased from Wenchang Baocheng Industry and Trade Co., Ltd. (Hainan, China). *n*-hexane, triethylamine (TEA), and trimethylchlorosilane (TMCS) were obtained from Aladdin Reagent Co., Ltd. (Shanghai, China). Sodium silicate was purchased from Macklin Biochemical Co., Ltd. (Shanghai, China). Sulfuric acid was bought from Yong Fei Chemical Reagent Co., Ltd. (Tianjin, China). All chemicals were of analytical grade and were used as received without any further purification.

### 4.2. Preparation of Bacterial Cellulose Matrix

**Figure 8:**
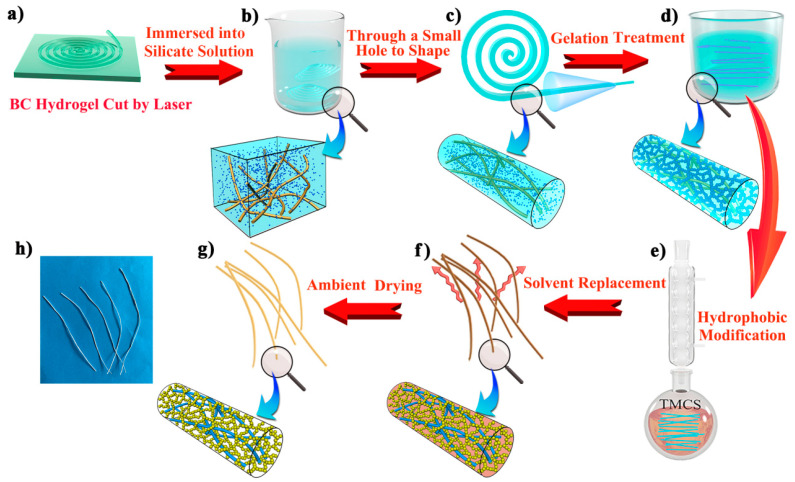
Schematic illustration of the preparation process of CAFs. A nata-de-coco slice was cut using a laser (**a**). The matrix was immersed in sodium silicate solution (**b**) and reshaped by a small hole mold (**c**). After the silica gel skeleton was formed in the matrix (**d**), CAFs were obtained through hydrophobic modification, solvent replacement, and ambient drying (**e**–**h**).

Nata-de-coco slices (i.e., bacterial cellulose hydrogel) of thickness 3.5 mm were repeatedly cleaned with deionized water to remove sugar. The washed nata-de-coco slices were heated to 90 °C in NaOH solution (4% *w*/*w*) for 6 h [43], then washed with deionized water until neutral. Next, the washed cellulose hydrogel was placed on a glass plate and most of the water was squeezed out (Figure S1a). Finally, a laser cutter (15 W power) was used to obtain a fiber-like BC matrix of uniform width (2 mm) and length (approximately 500 mm). This step is shown in Figure 8a and Figure S1b.

### 4.3. Preparation of Sodium Silicate Solution

Sodium silicate solutions with different concentrations were prepared using instant sodium silicate powder and deionized water. The specific dosages of the two substances are listed in Table 3.

### 4.4. Preparation of Silica-Bacterial Cellulose Composite Wet Gel Fibers

The wet fiber-like BC matrix was immersed in a sodium silicate solution. After sufficient diffusion for 2 h (Figure 8b and Figure S1c), the fiber-like BC matrix soaked in sodium silicate solution was scooped up and immediately passed through a tapered mold (1000 μL pipette tips with the front 2 cm removed were used) (Figure 8c and Figure S1d). Then, the fiber-like BC matrix containing the silica precursor became finer and more uniform. The molded fibers were soaked in 4 mol/L H_2_SO_4_ for 30 min (Figure 8d). With the diffusion of H^+^ into the fiber-like BC matrix as an acidic catalyst, the silicate in the matrix was transformed into a silica gel skeleton, and the gelatinous fibers were washed to neutral with deionized water to remove Na^+^, SO_4_^2−^, and excess H^+^, thus yielding silica-cellulose composite wet gel fibers.

### 4.5. Hydrophobic Modification and Atmospheric Drying of CAFs

The wet gel fibers were soaked in deionized water and heated at 70 °C for 1.5 h to make the gel skeleton more robust. Next, deionized water was replaced with ethanol for 3 h and then ethanol was replaced with *n*-hexane for 3 h for solvent replacement. Subsequently, *n*-hexane (50 mL), TEA (4 mL) (neutralized HCl to prevent cellulose from being hydrolyzed), and TMCS (3 mL) were added to the flask, and the wet gel fibers (approximately 4 g) were immersed in the solution. The flask was heated in an oil bath and refluxed for 2 h (Figure 8e). Afterward, the condensation reflux was complete. The wet gel fibers were immersed in a beaker containing ethanol which was replaced every 30 min and repeated twice to remove excess reagents and amine salts generated in the reaction. Then, ethanol was replaced with n-hexane, and the above operation was repeated. The hydrophobic modified wet gel fibers were heated in an oven at 80 °C for 20 min to obtain dry and hydrophobic CAFs (Figure 8g,h and Figure S1e).

### 4.6. Characterization

Thermal insulation test: first, several CAFs were packed tightly and aligned unidirectionally to form a single-layer mat approximately 0.7 mm thick and placed on a hot plate. The thermocouple was connected to the surface of the fiber and the hot plate, respectively, and the temperature change of the fiber surface (T_f_) was recorded when the hot plate (T_h_) was raised from room temperature to 200 °C. The absolute temperature difference between the fiber surface and the hot plate surface is denoted as (|ΔT|), with higher |ΔT| indicating better thermal insulation performance. To visually observe the differences in the surface temperatures of these fabrics, then (|ΔT|) with (T_h_) were analyzed.

The specific surface area, pore size distribution, and mechanical properties were determined, and the micromorphology, functional groups (FTIR), silica content of the CAFs, and wettability were examined. Finally the thermal insulation performance and thermal stability were evaluated. Detailed characterization methods are provided in the Supplementary Materials. 

## Figures and Tables

**Table 1:** Physical properties of CAFs.

Samples	SiO_2_ in Aerogels [% *w*/*w*]	Bulk Density [g cm^−3^]	S_BET_ [m^2^ g^−1^]	Pore Size [nm]	Porosity ^a^ [%]
CAF-1	22	0.080	158.5	13.2	80.3
CAF-2	38	0.123	404.7	13.0	89.1
CAF-3	46	0.146	548.1	13.7	90.6
CAF-4	55	0.154	671.3	9.5	90.2

^a^ Porosity includes the voids caused by crystal growth in the gel skeleton during gel freezing.

**Table 2:** Compositions, fabrication methods, tensile strength, and densities of the relevant aerogel fibers.

Materials	Fabrication Method	Tensile Strength	Density	Ref.
CA/PAA-SF	wet-spinning	2.6 ± 0.4 MPa	0.21 g/cm^3^	[19]
CA/PAA-SF/GO	coaxial wet-spinning	3.0 ± 0.2 MPa		[39]
QF/ASA	hydrothermal and Ti-H_2_O_2_	3.17 ± 0.04 MPa	0.302 g/cm^3^	[40]
PVA	freeze-spinning	8.36 MPa		[41]
SiO_2_-Cellulose	secondary shaping	5.4 MPa	0.164 g/cm^3^	[35]
SiO_2_	reaction-spun	230 KPa	0.15–0.2 g/cm^3^	[32]

**Table 3:** Sodium silicate solution of different concentrations formed by sodium silicate powder and deionized water.

Solution	SS-1	SS-2	SS-3	SS-4
Na_2_O·3SiO_2_ (g)	10	22	30	50
H_2_O (mL)	170	178	150	180
SiO_2_ in solution (% *w*/*w*)	4	8	12	16
Corresponding sample number	CAF-1	CAF-2	CAF-3	CAF-4

## Data Availability

The data presented in this study are available on request from the corresponding author.

## Supplementary Materials

The following are available online at https://www.mdpi.com/article/10.3390/gels8010017/s1, Video S1: Video of the three-point bending test of the CAF-2, Figure S1: Photos of the preparation process of CAFs; Figure S2: Thermal insulation properties of CAF-3 fabric with different layers; Figure S3: (**a**) Thermogravimetry analysis (TGA, 10 °C min^−1^ heating) curves of BC matrix, cotton thread, and silk fabric. (**b**) Thermogravimetry analysis (TGA, 10 °C min^−1^ heating) curves of BC matrix and CAFs; Figure S4: ATR-FTIR spectra of HSAs, HBC, and CAF-3; Characterization and Reference.
