# Supplementary material for: Silica-Bacterial Cellulose Composite Aerogel Fibers with Excellent Mechanical Properties from Sodium Silicate Precursor
Source: Gels. 2021 Dec 26;8(1):17. doi: 10.3390/gels8010017 (PMC8774922; doi:10.3390/gels8010017)
Supplement: Supplementary file 1 [file gels-08-00017-s001.zip › Supplementary Materials/gels-1517755-supplementary.pdf]

gels

MDPI

Supplementary

Silica-Bacterial Cellulose Composite Aerogel Fibers with Excellent Mechanical Properties from Sodium Silicate Precursor

Qiqi Song [1,2,3,†], Changqing Miao [1,2,3,†], Huazheng Sai [1,2,3,*], Jie Gu [1,2,3], Meijuan Wang [1,2,3], Pengjie Jiang [1,2,3], Yutong Wang [1,2,3], Rui Fu [1,2,3,*] and Yaxiong Wang [1,2,3]

[1] School of Chemistry and Chemical Engineering, Inner Mongolia University of Science & Technology, Baotou 014010, China; songqiqiaa@163.com (Q.S.); qingmc@163.com (C.M.); gujie199504182021@163.com (J.G.); wmjbest1014@163.com (M.W.); jpj1692787089@163.com (P.J.); wangyut@163.com (Yutong Wang); wangyaxiong2021@126.com (Yaxiong Wang)
[2] Inner Mongolia Engineering Research Center of Comprehensive Utilization of Bio-Coal Chemical Industry, Inner Mongolia University of Science & Technology, Baotou 014010, China
[3] Aerogel Functional Nanomaterials Laboratory, Inner Mongolia University of Science & Technology, Baotou 014010, China
* Correspondence: shz15@tsinghua.org.cn (H.S.); furui14@mails.ucas.edu.cn (R.F.)
† These two authors contributed equally to this work.

1. Supplementary Video

Video S1
Video of the three-point bending test of the CAF-2.

2. Characterization

2.1. Characterization on Micromorphology

The micromorphology of the products were observed using scanning electron microscopy (SEM, SIGMA500, ZEISS, Germany) at an acceleration voltage of 3 kV after sputtering with gold for its porous structure and insulation.

2.2. Characterization on Mechanical Properties

The mechanical properties of the fibers were tested by an electronic universal testing machine (HD-B609B-S, Guangdong, China), in the tensile mode. The strain rate was 2 mm/min for the tests. All the test samples were 2 cm. Each set of tensile strength test results are collected from at least five samples to obtain reliable values. The force was loaded at a speed of 2 mm/min for the three-point bending tests with a fixture span of 15 mm.

2.3. Density Measurement

Take the CAFs sample of 8 cm length, combined with the diameter given by SEM images to calculate the total volume of the sample (v_c). Its mass (m_c) was measured by a balance with a precision of 0.0001 g. The density of CAFs is calculated according to the ratio of mass to volume (m_c/v_c).

2.4. Nitrogen Physisorption Measurement

The specific surface aera of the fibers were measured by the Brunauer-Emmett-Teller (BET) method in the condition of the Nitrogen adsorption at pressures $0.01 < p/p_0 < 1.0$. The Barrett-Joyner-Halenda (BJH) Analyses were conducted from desorption isotherm when the pore-size distribution was investigated. Content of silica in the CAFs: The dried BC matrix of 5 cm in length was intercepted, its mass was weighed (m_0). Mass fraction (ω_s) of silica could be calculated by the equation:

$$\text{Mass fraction } (\omega_s) = \frac{m_c - m_0}{m_c} \quad \text{(S1)}$$

2.5. Porosity Measurement

The porosity of CAFs was calculated according to Equation S2, where ρ_s and ρ_c are the bulk density of CAFs, the skeleton densities of pure silica aerogels and biopolymer (i.e. BC) matrix; ω_s and ω_c were the mass fraction of silica and BC in CAFs, respectively. Herein, based on literature data, the ρ_s and ρ_c were designed as 2.1 g cm^{-3} and 1.59 g cm^{-3}[1].

$$\text{Porosity } (\%) = \left(1 - \frac{\rho}{\omega_s \rho_s + \omega_c \rho_c}\right) \times \% \quad \text{(S2)}$$

2.6. Thermal Insulation Measurement

The thermographs were obtained by a thermal infrared camera (FLIR T620, Teledyne FLIR, Woburn, MA, USA). The camera was operated at a distance of about 30 cm. The CAFs sample were put on the heating plate, one thermo couple was connected to the sample and the other was on the hotplate next to the sample. Gradually heating up, the date acquisition device (34972A, Agilent, Santa Clara, CA, USA) was used to record the temperature of two thermo couples simultaneously.

2.7. Wettability Test

The wettability of the sample was tested by video optical contact angle measuring instrument (OCA25L, DataPhysics Instruments GmbH, Filderstadt, Germany). Firstly, about 3 μL of deionized water was extruded from the syringe and hung at the tip of the needle. The wettability of the sample was observed during the process as it contacts the water droplet and then leaves the water droplet

2.8. Fourier Transform Infrared (FTIR) Spectroscopy

The attenuated total reflection (ATR) infrared spectra of HSAs, HBC and CAF-3 were obtained on a VERTEX 70 FT-IR spectrometer (Bruker, Germany). All spectra were recorded between 3600 and 400 cm^{-1} with a resolution of 4 cm^{-1} and 16 scans per sample.

3. The Photos of the Preparation Process of CAFs

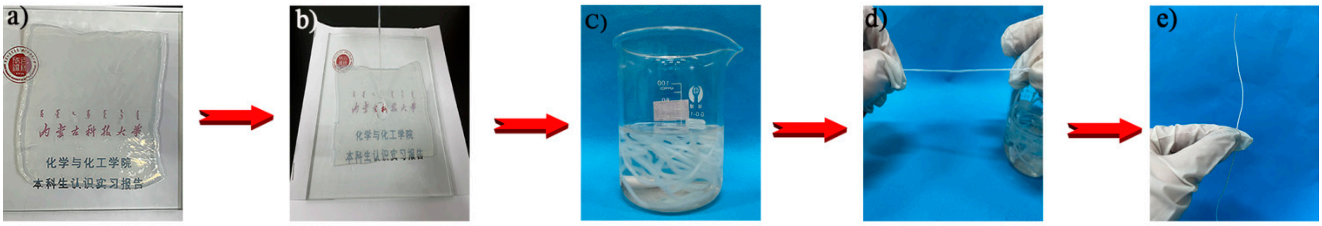

Figure S1. The photos of the preparation process of CAFs: (**a**) BC hydrogel slice, (**b**) fiber-like BC hydrogel, (**c**) the immersion of BC in sodium silicate solution, (**d**) the secondary shaping of the BC matrix containing silica precursor, (**e**) the sample of CAF.

4. Thermal Insulation Properties of CAF-3 Fabric with Different Layers

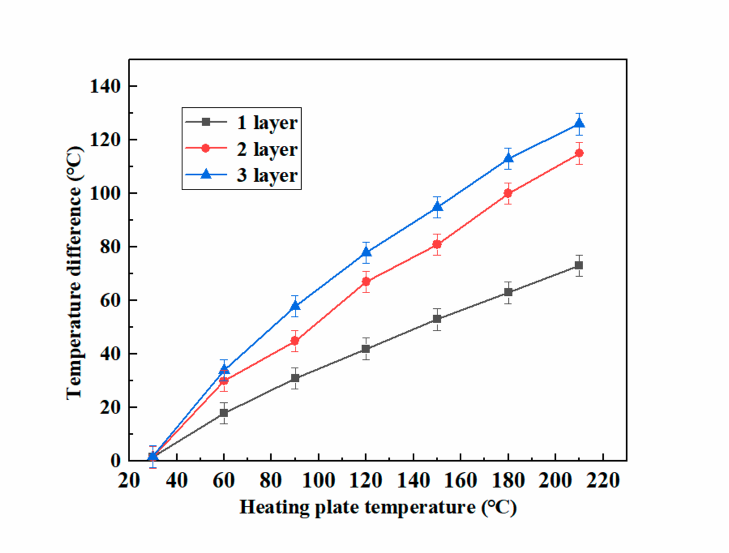

Figure S2. Thermal insulation properties of CAF-3 fabric with different layers.

5. Thermal Stability

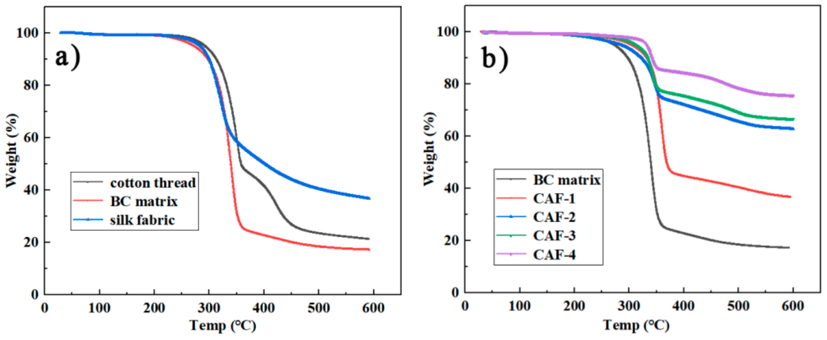

Figure S3. (**a**)Thermogravimetry analysis (TGA, 10 °C min^{-1} heating) curves of BC matrix, cotton thread, and silk fabric. (**b**) Thermogravimetry analysis (TGA, 10 °C min^{-1} heating) curves of BC matrix and CAFs.

6. Infrared Spectroscopy

As shown in Figure S4, hydrophobic silica aerogels (HSAs) contained abundant Si-O-Si bonds, but there was no Si–O–Si bond in hydrophobic bacterial cellulose (HBC) [2]. Therefore, Si–O–Si bond could be used as a basis for judging whether silica was successfully combined with bacterial cellulose. Both HSAs and CAF-3 could be found at Si–O–Si and Si–CH_3 stretching vibration at 827cm^{-1} and 847 cm^{-1} [3,4]. In HBC, Si–CH_3 stretching vibration was found only at 845 cm^{-1}. In addition, the C–O stretching vibration of HBC at 1107 cm^{-1}, and the C–O stretching vibration was also reflected in CAF-3. In conclusion, infrared spectroscopy analysis proved that CAFs had been successfully prepared [5,6].

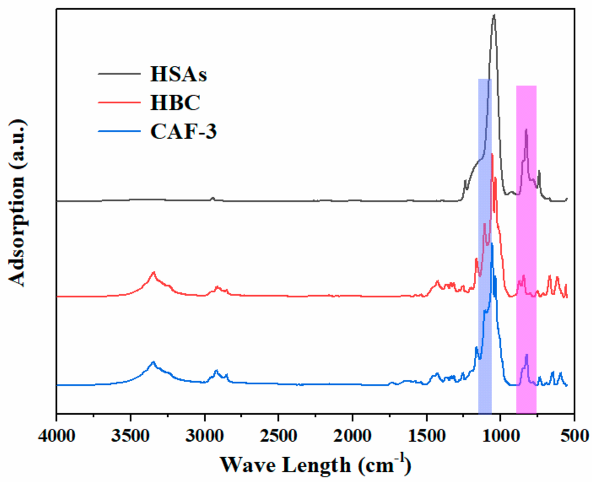

Figure S4. ATR-FTIR spectra of HSAs, HBC, and CAF-3.

Reference

1. Heath, L., Thielemans, W. Cellulose nanowhisker aerogels. *Green Chem.* **2010**, *12*, 1448–1453.
2. Dourbash, A.; Motahari, S.; Omranpour, H. Effect of water content on properties of one-step catalyzed silica aerogels via ambient pressure drying. *J. Non-Cryst. Solids* **2014**, *405*, 135–140.
3. Sai, H.Z.; Fu, R.; Xing, L.; Xiang, J.H.; Li, Z.Y.; Li, F.; Zhang, T. Surface Modification of Bacterial Cellulose Aerogels' Web-like Skeleton for Oil/Water Separation. *ACS Appl. Mater. Interfaces* **2015**, *7 (13)*, 7373–7381.
4. Qin, H.; Zhang, Y.; Jiang, J.; Wang, L.; Song, M.; Bi, R.; Zhu, P.; Jiang, F. Multifunctional Superelastic Cellulose Nanofibrils Aerogel by Dual Ice-Templating Assembly. *Adv. Funct. Mater.* **2021**, *31 (46)*, 2106269.
5. Zhang, Z.; Sèbe, G.; Rentsch, D.; Zimmermann, T.; Tingaut, P. Ultralightweight and Flexible Silylated Nanocellulose Sponges for the Selective Removal of Oil from Water. *Chem. Mater.* **2014**, *26*, 2659–2668.
6. Taajamaa, L.; Kontturi, E.; Laine, J.; Rojas, O.J. Bicomponent Fibre Mats with Adhesive Ultra-Hydrophobicity Tailored with Cellulose Derivatives. *J. Mater. Chem.* **2012**, *22*, 12072−12082.
